# Central Nervous System Inflammation Induced by Lipopolysaccharide Up-Regulates Hepatic Hepcidin Expression by Activating the IL-6/JAK2/STAT3 Pathway in Mice

**DOI:** 10.3389/fnut.2021.649640

**Published:** 2021-04-01

**Authors:** Fali Zhang, Peng Zhao, Zhongming Qian, Mingkang Zhong

**Affiliations:** ^1^Department of Pharmacy, Huashan Hospital, Fudan University, Shanghai, China; ^2^Institute of Translational & Precision Medicine, Laboratory of Neuropharmacology, Nantong University, Nantong, China; ^3^Laboratory of Neuropharmacology, National Clinical Research Center for Aging and Medicine, Huashan Hospital, Fudan University, Shanghai, China

**Keywords:** AG490, stat3, IL-6-/-mice, LPS, hepcidin

## Abstract

It is known that lipopolysaccharide (LPS) triggers inflammatory response after intracerebroventricular (ICV) injection and elevates the expression of hepcidin through the interleukin 6/janus kinase 2/transducer and activator of the transcription 3 (IL-6/JAK2/STAT3) signaling pathway in the brain. This study was conducted to determine whether LPS ICV injection can regulate peripheral hepatic hepcidin expression and iron metabolism. Here, we studied the hepcidin expression in the liver, as well as serum iron and transferrin saturation, after LPS ICV injection. We also demonstrated the role of the IL-6/JAK2/STAT3 pathway in hepcidin expression in the livers of IL-6 knockout (IL-6–/– mice) and IL-6+/+ mice. AG490 was used to verify the effect of the IL-6/JAK2/STAT3 pathway on hepatic hepcidin expression. Our present study demonstrated that LPS ICV injection up-regulated hepatic hepcidin expression. This finding provides further evidence for highlighting the importance of the central inflammation on hepatic hepcidin expression and peripheral iron metabolism.

## Introduction

The brain is protected by the blood–brain barrier (BBB) when peripheral tissues are infected (1). This protection can be impaired by a defect or dysfunction in the BBB. Lipopolysaccharide (LPS)-triggered inflammatory reaction by the injection into the lateral ventricle of the brain is induced by the activation of astrocyte and microglia cells in the brain and the release of inflammatory cytokines (2). Several studies have demonstrated that central nervous system (CNS) infections could induce peripheral inflammatory reactions, thereby leading to the production of inflammatory cytokines (3). The underlying mechanisms that are involved in peripheral inflammatory reaction because of CNS infection mainly include the hypothalamic–adrenal–pituitary (HPA) axis, the sympathetic nervous system, inflammatory cytokines, and reactive oxygen species (ROS) (4, 5). LPS is not restricted in the brain when injected into the lateral ventricle. Previous research has shown that intracerebroventricular (ICV) injection of LPS into mice increased BBB permeability 4 h after injection, which subsequently results in LPS leakage into peripheral circulation (6).

Iron is necessary for many metabolic processes in the central and peripheral nervous systems, acting as the electron transfer and co-factor for many metabolic enzymes (7). However, abnormally high levels of iron generate ROS and enhance oxidative stress, resulting in cell death. Therefore, it is important to maintain normal iron levels and iron homeostasis to ensure metabolic balance and normal physiological function (8). It has been well-demonstrated that hepatic hepcidin is the key mediator in iron homeostasis and iron metabolism disorders (9, 10). Hepcidin induces the degradation of ferroportin (Fpn1) protein and subsequently inhibits the release of iron into plasma from duodenal enterocytes (11, 12). Therefore, hepcidin inhibits the absorption of dietary iron, and the recycling of iron from splenic and liver macrophages and senescent erythrocytes, as well as hepatocytes (13, 14). Hereditary hemochromatosis with iron overload is usually associated with hepcidin deficiency. However, elevated expression of hepcidin in inflammatory diseases contributes to the development of anemia with iron restricted. To date, the regulation of hepcidin is found to be transcriptional. Iron status and inflammation in the body are investigated to increase hepcidin expression in liver through the BMP/SMAD signaling pathway. Nevertheless, the JAK2/STAT3 pathway is known as the major pathway in the elevation of hepcidin transcription stimulated by inflammatory cytokines (15, 16). LPS generates many inflammatory cytokines by recognizing specific TLR's families and subsequently stimulates acute hypoferremia mainly due to elevated expression of hepcidin in the liver (17). Several cytokines including primarily IL-6, IL-1, IL-22, and interferon α (18–21) have been found to up-regulate hepcidin expression mediated by the JAK2/STAT3 pathway (22, 23).

In our previous study, we have demonstrated that LPS increased hepcidin expression in neurons mediated by the activation of the IL-6/STAT3 signaling pathway in microglia cells (24). IL-6 is a multifunctional cytokine that regulates various aspects of the immune response, acute-phase reaction, and the expression of hepcidin in response to inflammation (25–27). Our previous *in vivo* study also showed that IL-6 knockout significantly decreased hepcidin expression and STAT3 phosphorylation in the cortex and hippocampus, as well as Fpn1 and ferritin light chain (Ft-L) with LPS ICV injection (28). However, whether the CNS infection, induced by LPS ICV injection, affected peripheral iron metabolism was unknown. In this study, we demonstrated the effect of LPS ICV injection on serum iron levels and hepcidin expression in the liver. Then, IL-6/JAK2/STAT3 signaling pathway was also studied for hepcidin regulation on IL-6 knockout mice (IL-6–/–). In addition, AG490 was used as the inhibitor of JAK2 to verify the activation of the IL-6/JAK2/STAT3 signaling pathway in response to LPS ICV injection. The present *in vivo* findings suggest that the liver hepcidin expression and iron metabolism are regulated by CNS infection.

## Materials and Methods

### Mice and Chemicals

The animal care and experimental procedures were performed according to the Animal Management Rules of the Ministry of Health of the People's Republic of China and approved by the Animal Ethics Committees of Fudan University, Shanghai, China. Wild-type mice (C57BL/6, 8-week-old, body weight ~25 g) and IL-6 knockout mice (IL6–/–, provided by Jackson Laboratories, Bar Harbor, Maine, USA) were supplied with water and food *ad libitum* and housed under the 12-h light–dark cycle. The JAK2 inhibitor AG490 was purchased from Selleckchem (Houston, TX, USA); the other chemicals were obtained from Sigma-Aldrich (St. Louis, MO, USA).

### Lipopolysaccharide Administration by Intracerebroventricular Injection

LPS administration by ICV injection was performed as previously described (29). LPS (*Escherichia coli* 055: B5; 5 μg in 2 μL of sterile saline) or vehicle (sterile saline, 2 μL) was injected into the cerebral lateral ventricle using a 10-μL syringe with a 33-gauge needle at a rate of 0.5 μL/min. The stereotaxic coordinates of ICV injection were −3.0 mm dorsal/ventral, −1.0 mm lateral, and −0.5 mm anterior/posterior from the bregma. After the injection, the needle was kept for an additional 5 min. Liver tissues were harvested for analysis at 6 h after LPS ICV injection.

### Enzyme-Linked Immunosorbent Assay

The concentration of IL-6 was determined using IL-6 ELISA kits (R&D Systems, Minneapolis, MN, USA) in accordance with manufacturer's instructions. Briefly, liver tissue was digested and homogenized using radioimmunoprecipitation assay (RIPA) lysis buffer (Beyotime, Shanghai, China). Tissue lysis solution was centrifuged at 12,000 × g and 4°C for 15 min, and then supernatant solution was collected for protein concentration detection and ELISA.

### Real-Time Quantitative PCR

Total RNA was extracted using TRIzol reagent, and cDNA preparation was performed using Revert Aid First Strand cDNA Synthesis Kit (Thermo Fisher, Shanghai, China) in accordance with manufacturer instructions. Real-time PCR was conducted using FastStart Universal SYBR Green Master and LightCycler^®^ 96 instrument (Roche, Switzerland). Quantitative calculations were based on CT values of each target gene. Results were normalized to β-actin levels. Relative fold of gene expression compared with control was calculated using the 2^−ΔΔ*CT*^ method. Primers for IL-6 were provided by Primer Bank. Mouse IL-6: forward, 5′-*CTGCAAGAGACTTCCATCCAG*-3′ and reverse, 5′-*AGTGGTATAGACAGGTCTGTTGG*-3′; mouse hepcidin: forward, 5′-*AGAGCTGCAGCCTTTGCAC*-3′ and reverse, 5′-*GAAGATGCAGATGGGGAAGT*-3′; mouse β-actin: forward, 5′-*AAATCGTGCGTGACATCAAAGA*-3′ and reverse, 5′-*GCCATCTCCTGCTCGAAGTC*-3′.

### Western Blot

The tissues were homogenized in RIPA lysis buffer and then sonicated on ice as described previously (29). Bicinchoninic acid (BCA) (Pierce, Rockford) method was used to detect protein concentrations. Equivalent amounts of protein (30–40 g) were loaded in each sample well. The blots were blocked with skim milk and then incubated with the primary antibodies overnight at 4°C: anti-pSTAT3 (rabbit polyclonal, 1:1,000, Cell Signaling Technology), anti-STAT3 (mouse monoclonal, 1:1,000, Cell Signaling Technology), anti-Fpn1 (rabbit polyclonal, 1:1,000, Novus), anti-Ferritin-L (rabbit polyclonal, 1:1,000, ProteinTech), and anti-β-actin (mouse monoclonal, 1:10,000, Sigma). The blots were then washed in phosphate-buffered saline (PBS) three times and incubated with secondary antibody for 2 h (goat anti-rabbit IRDye 800 CW, 1:1,000; goat anti-mouse IRDye 800 CW, 1:5,000). The intensities of the bands were analyzed using the Odyssey Imaging System (Li-Cor, NE, USA).

### Serum Iron and Transferrin Saturation Assay

One percent pentobarbital sodium was used to anesthetize mice [40 mg/kg body weight, intraperitoneal (IP) injection]. Blood samples were obtained from the abdominal aorta using anticoagulant syringes. Commercial kits were used to measure serum iron (SI) and unsaturated iron binding capacity (UIBC) as previously described (29). Then, total iron binding capacity (TIBC; TIBC = serum iron + UIBC) and transferrin saturation (TS; TS = SI/TIBC × 100) were calculated and analyzed.

### Statistical Analyses

GraphPad Prism was used to conduct the statistical analyses. Differences between means ± SEM in each of the two groups were calculated using the two-tailed Student's *t* test. Differences between more than two groups were compared using one-way or two-way analysis of variance (ANOVA) followed by the Sidak *post hoc* test. All *p-*values were two-sided, and *p* < 0.05 was considered statistically significant.

## Results

### Hepcidin mRNA, IL-6 mRNA, and Protein Expression in the Liver of IL-6–/– Mice Were Significantly Lower Than Those in IL-6+/+ Mice After Lipopolysaccharide Administration

**Figure 1:**
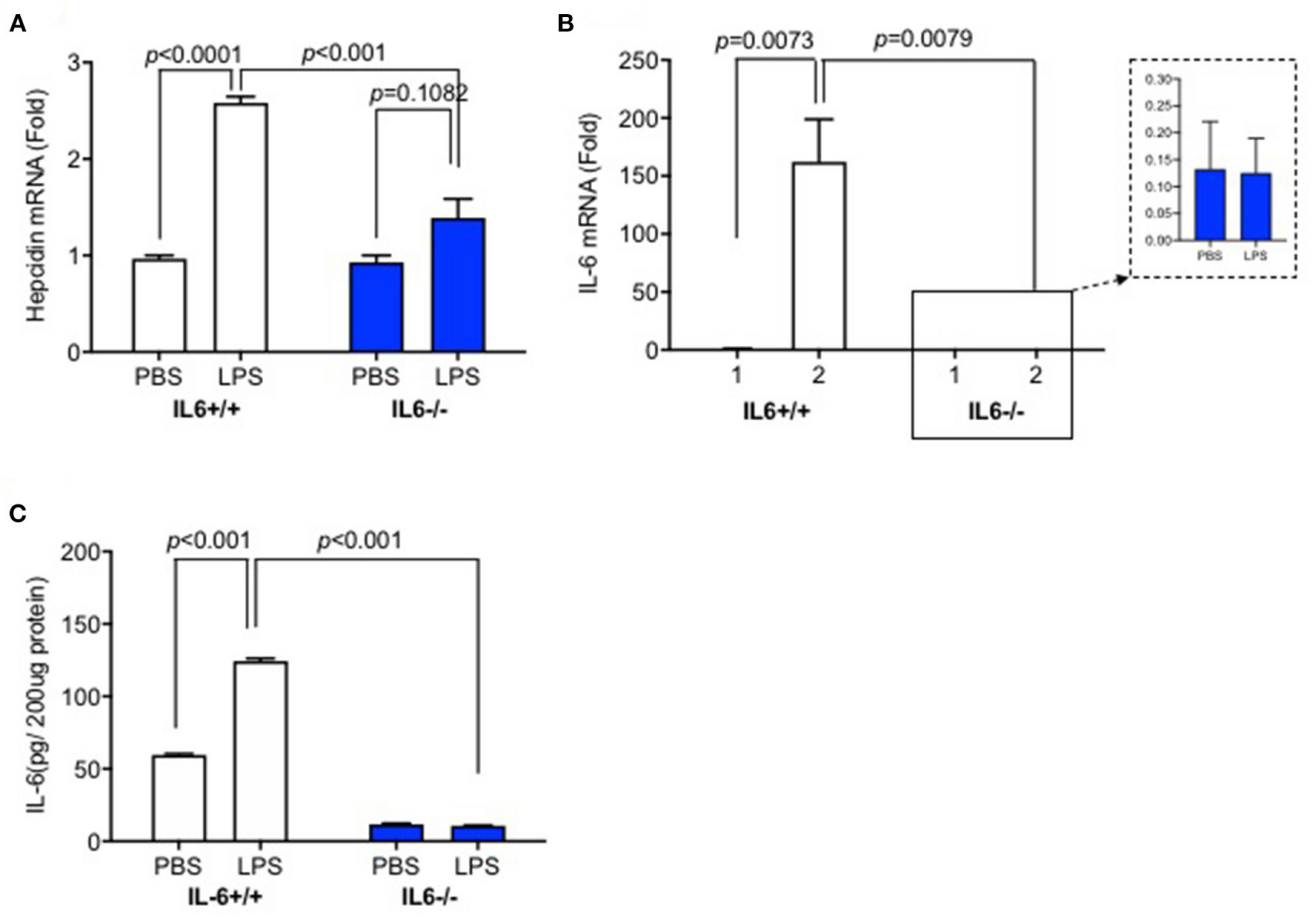
Hepcidin mRNA, IL-6 mRNA, and protein expression in the liver of IL-6–/– mice were significantly lower than those in IL-6+/+ mice after lipopolysaccharide (LPS) administration. IL-6+/+ and IL6–/– mice received intracerebroventricular (ICV) injection of LPS (5 μg). After 6 h, the expression of hepcidin mRNA **(A)** and IL-6 mRNA **(B)** was detected using RT-PCR, and IL-6 protein expression **(C)** was measured using ELISA. Data are expressed as the means ± SEM (% control) (IL-6+/+: PBS = 4, LPS = 6; IL-6–/–: PBS = 3, LPS = 4). Analysis between the treatments was done via two-way ANOVA with Sidak's *post hoc* test.

Based on our previous study, which showed that hepcidin mRNA in the brain was significantly higher after LPS ICV injection, we first investigated the expression levels of hepcidin and IL-6 in the liver when mice were administered with ICV injection of LPS. We detected hepcidin mRNA, IL-6 mRNA, and protein expression at 6 h after LPS ICV injection. The results showed that hepcidin mRNA (Figure 1A, *p* < 0.0001), IL-6 mRNA (Figure 1B, *p* = 0.0073), and protein expression (Figure 1C, *p* < 0.001) significantly increased in the liver of IL-6+/+ mice with LPS ICV injection, but not for the IL-6–/– mice. There was a significant interaction between group and treatment for hepcidin mRNA was observed [*p* = 0.0003, *F*_(1, 12)_ = 24.89], for IL-6 mRNA [*p* = 0.0073, *F*_(1, 14)_ = 9.850] and also for IL-6 protein [*p* < 0.0001, *F*_(1, 8)_ = 504.1]. A significant difference was observed for hepcidin mRNA (*p* < 0.001) between the group after LPS treatment. There was also significant difference between group for IL-6 mRNA (*p* = 0.0079) and IL-6 protein (*p* < 0.001) after LPS treatment. In addition, IL-6–/– mice displayed a slightly higher expression of hepcidin mRNA in the liver after LPS injection, although there was no significant difference between LPS and PBS-treated IL-6–/– mice (Figure 1A, *p* = 0.1082). These findings indicate that inflammatory reaction in the brain from LPS ICV injection can affect hepatic hepcidin mRNA and IL-6 mRNA expression, and IL-6 knockout abolished hepcidin expression induced by LPS; this result claimed that IL-6 was the inducer of hepcidin expression.

### IL-6 Deficiency Diminished the Decrease of Serum Iron and Transferrin Saturation Induced by Lipopolysaccharide Injection

**Figure 2:**
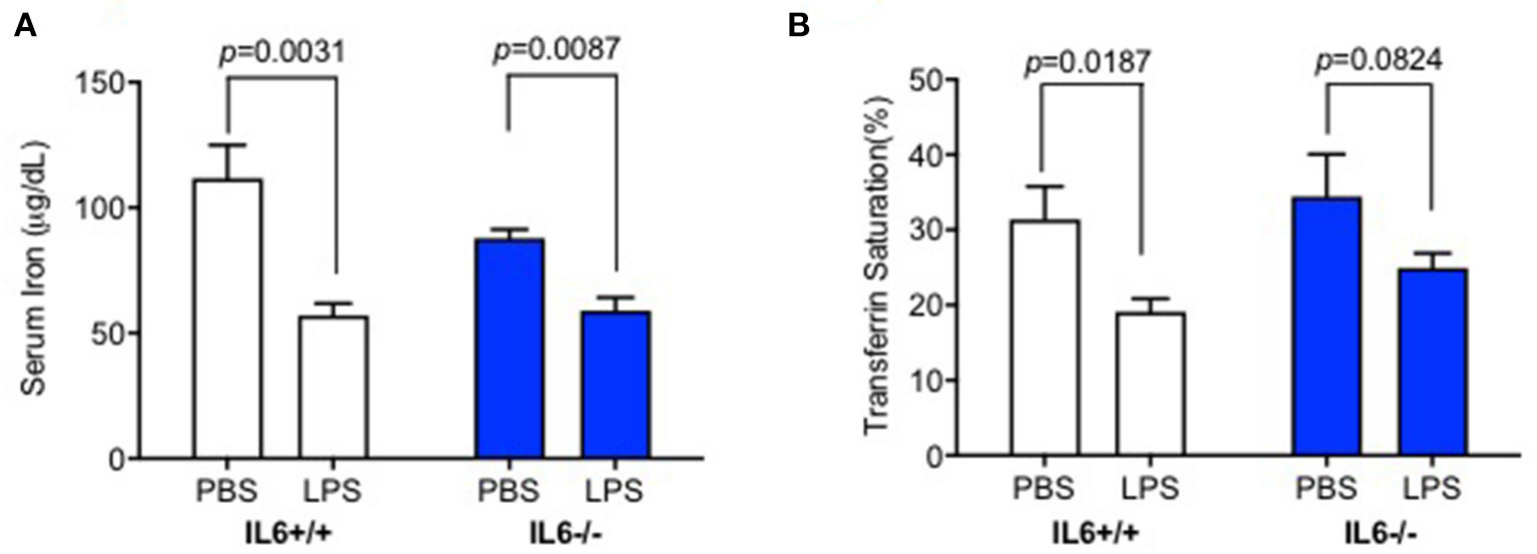
IL-6 deficiency diminished the decrease of serum iron and transferrin saturation induced by lipopolysaccharide (LPS) injection. IL-6+/+ and IL6–/– mice were administered with phosphate-buffered saline (PBS) or LPS intracerebroventricular (ICV) injection and sacrificed after 6 h; serum iron concentration **(A)** and transferrin saturation **(B)** were measured. Data are presented as means ± SEM (% control) (IL-6+/+: PBS = 4, LPS = 6; IL-6–/–: PBS = 3, LPS = 4). Analysis between the treatments was done via two-way ANOVA with Sidak's *post hoc* test.

We investigated whether LPS treatment by ICV injection could affect peripheral iron metabolism. Serum iron and transferrin saturation assays were conducted to confirm the effects of elevated hepcidin expression in the liver of IL-6+/+ mice. There was no significant interaction between group and treatment for serum iron [*p* = 0.1793, *F*_(1, 17)_ = 1.962] or transferrin saturation [*p* = 0.6828, *F*_(1, 18)_ = 0.1725]. Serum iron (Figure 2A, *p* = 0.0031) and transferrin saturation (Figure 2B, *p* = 0.0187) significantly decreased in IL-6+/+ mice after LPS treatment; however, no statistically significant differences were observed for transferrin saturation (Figure 2B, *p* = 0.0824) in IL-6–/– mice. Serum iron in LPS-treated mice was slightly lower than those in PBS-treated IL-6–/– mice, with significant difference (Figure 2A, *p* = 0.0087); this result indicated that other cytokines could affect the serum iron, and we needed more further investigations to clarify this. Our results implied that the enhanced inflammation in the CNS could decrease peripheral serum iron and transferrin saturation; however, IL-6 knockout abolished the changes. These data indicated that IL-6 could affect the peripheral iron metabolism perhaps by inducing hepcidin expression.

### IL-6 Deficiency Eliminated the Decrease of Fpn1 and the Increase of p-STAT3 and Ft-L Protein Induced by Lipopolysaccharide in the Liver

**Figure 3:**
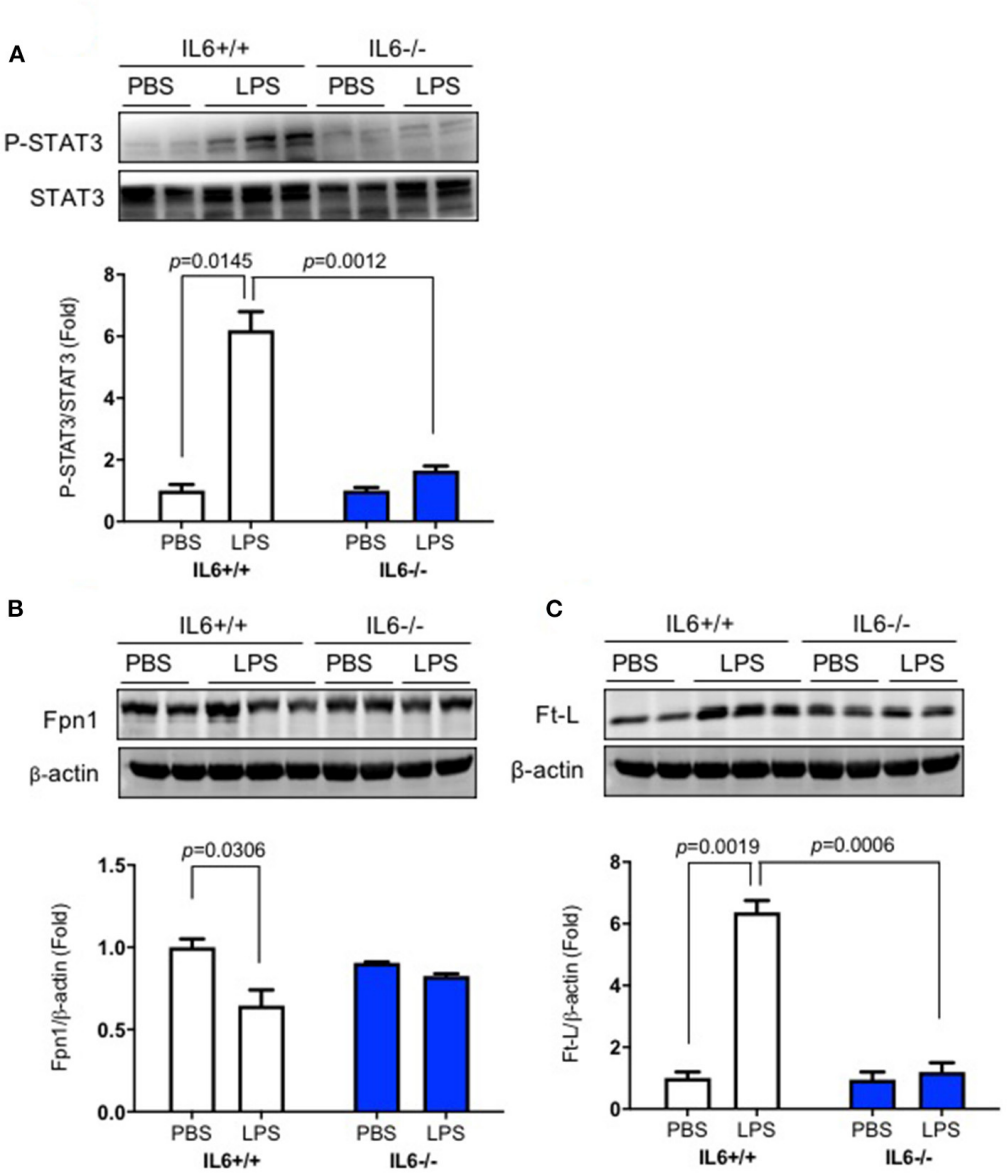
IL-6 deficiency eliminated the decrease of Fpn1 and the increase of p-STAT3 and Ft-L protein induced by lipopolysaccharide (LPS) in the liver. IL-6+/+ and IL6–/– mice were administered with phosphate-buffered saline (PBS) or LPS intracerebroventricular (ICV) injection. At 6 h after LPS treatment, p-STAT3 **(A)**, Fpn1 **(B)**, and Ft-L **(C)** protein expression levels in the liver were detected using western blot. Data are presented as means ± SEM (% control) (IL-6+/+: PBS = 3, LPS = 6; IL-6–/–: PBS = 3, LPS = 3). Analysis between the treatments was done via two-way ANOVA with Sidak's *post hoc* test.

To determine the mechanisms involved in hepatic hepcidin expression after LPS ICV injection, we examined phosphorylated STAT3 protein expression in the liver. We found that significant interaction between group and treatment for p-STAT3 [*p* = 0.0023, *F*_(1, 4)_ = 47.87] and Ft-L protein [*p* = 0.0007, *F*_(1, 5)_ = 56.15], but not for Fpn1 protein [*p* = 0.0644, *F*_(1, 4)_ = 6.423]. There was a significant difference for p-STAT3/STAT3 levels in the liver of LPS-treated IL-6+/+ mice (Figure 3A, *p* = 0.0145); the same changes were also observed for Fpn1 (Figure 3B, *p* = 0.0306) and Ft-L protein (Figure 3C, *p* = 0.0019); however, IL-6 deficiency diminished these changes in the liver of IL-6–/– mice after LPS treatment. A significant difference was observed between the groups for p-STAT3 (Figure 3A, *p* = 0.0012) and Ft-L protein (Figure 3C, *p* = 0.0006) after LPS treatment. These findings implied that LPS ICV injection up-regulated hepcidin expression through the IL-6/STAT3 signaling pathway. As shown in previous studies, hepcidin could bind to Fpn1, thereby inducing its internalization (10, 21), and the elevation of Ft-L protein was a marker of increasing tissue iron content (30). We also investigated the expression of Fpn1 and Ft-L protein in the liver. The results showed a significant reduction in the expression of Fpn1 protein and an increased expression of Ft-L protein in IL-6+/+ mice after LPS treatment, but such changes were not found in the liver of IL-6–/– mice. In addition, the expression levels of Fpn1 and Ft-L protein in the liver of IL-6+/+ mice did not differ from those in the liver of IL-6–/– mice with PBS treatment. This finding indicated that IL-6 induced by central LPS administration also affected the liver iron metabolism through STAT3 activation, and IL-6 could influence the liver expression of Fpn1 and Ft-L protein.

### AG490 Significantly Reduced p-STAT3 Protein Expression and Eliminated the Changes in Fpn1 and Ft-L Expression Induced by Lipopolysaccharide in the Liver

**Figure 4:**
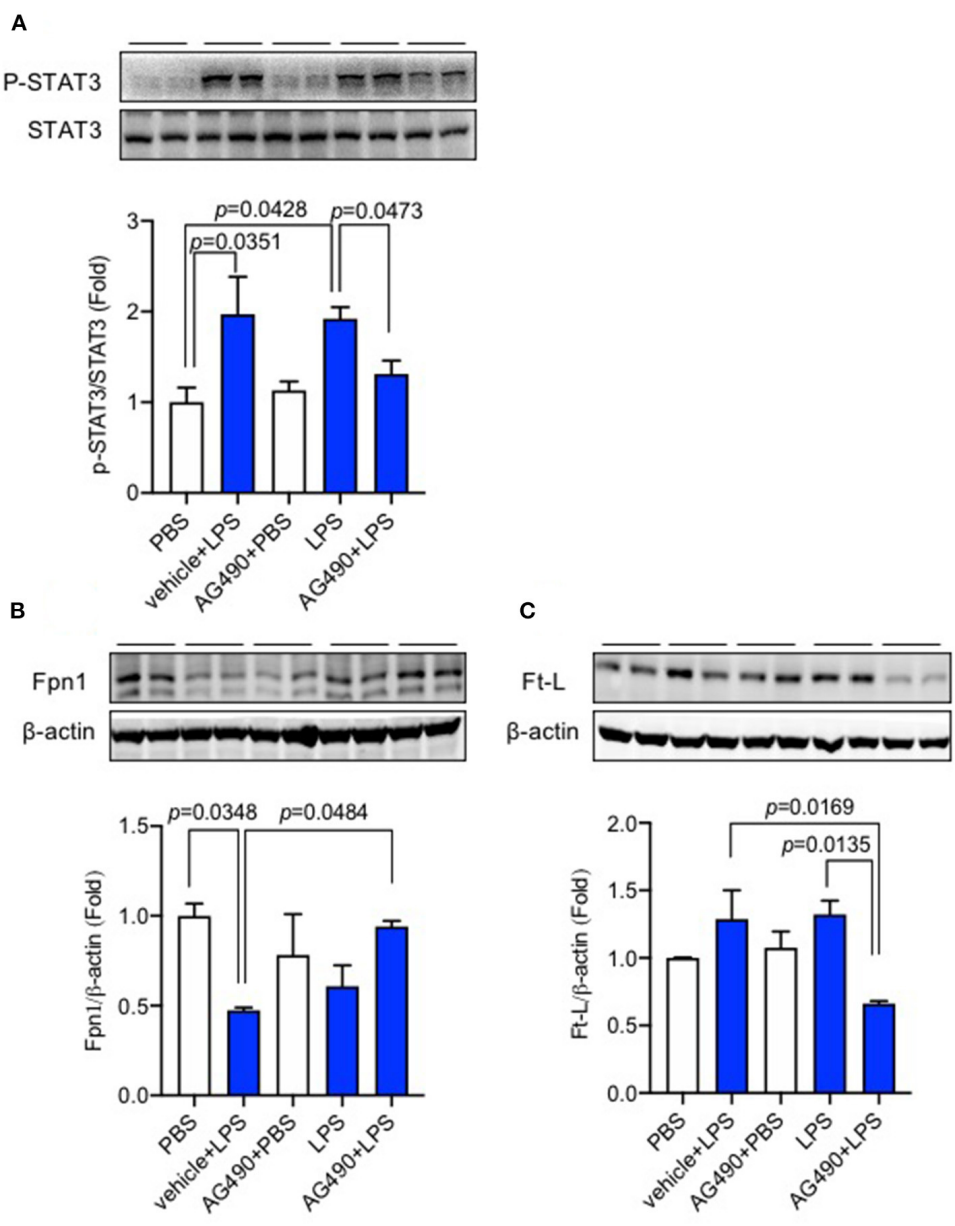
AG490 significantly reduced p-STAT3 protein expression and eliminated the changes in Fpn1 and Ft-L expression induced by lipopolysaccharide (LPS) in the liver. C57BL/6 wild-type male mice were pre-treated with AG490 (5 mg/kg, IP injection) in 10% dimethyl sulfoxide (DMSO) or vehicle 30 min before LPS (ICV injection, 5 μg in 2 μL of sterile saline). The expression levels of p-STAT3 **(A)**, Fpn1 **(B)**, and Ft-L proteins **(C)** in the liver were measured using western blot. Data are presented as means ± SEM (% control) (*n* = 4). For statistical analysis, one-way ANOVA with Tukey's *post hoc* test was performed.

Finally, we demonstrated the role of the IL-6/JAK2/STAT3 signaling pathway on hepatic hepcidin expression. AG490 as an inhibitor of JAK2 and the IL-6/JAK2/STAT3 pathway was pre-injected (IP injection) into C57BL/6 wild-type mice before LPS ICV injection; data showed that the level of p-STAT3/STAT3 was dramatically increased after LPS treatment (Figure 4A, *p* = 0.0351, *p* = 0.0428), and AG490 inhibited this increase of p-STAT3/STAT3 (Figure 4A, *p* = 0.0473). We also investigated the effect of AG490 on Fpn1 and Ft-L protein expression levels in the liver, and a significant difference between vehicle + LPS and AG490 + LPS groups was observed for Fpn1 protein (Figure 4B, *p* = 0.0484) and for Ft-L protein (Figure 4C, *p* = 0.0169). We found that inhibiting the IL-6/JAK2/STAT3 pathway with AG490 significantly eliminated the decrease of Fpn1 and increase of Ft-L protein induced by LPS treatment. This finding demonstrated that the activation of the IL-6/JAK2/STAT3 signaling pathway promoted the hepcidin expression in the liver after LPS ICV injection, and IL-6 affected the liver expression of Fpn1 and Ft-L protein through JAK2/STAT3 pathway.

## Discussion

The primary objective of this study was to investigate the effects of CNS inflammation, which was induced by LPS ICV injection, on hepcidin expression and iron metabolism in the liver, and to determine whether the IL-6/JAK2/STAT3 signaling pathway in the liver activated the hepcidin expression after LPS injection in the brain. In the present study, we demonstrated that hepcidin and IL-6 mRNA, as well as phosphorylation of STAT3, increased significantly after LPS ICV injection, and elevated hepcidin expression led to decreased Fpn1 protein and increased Ft-L protein expression levels in the liver. However, IL-6 knockout remarkably diminished hepcidin mRNA expression and suppressed the IL-6/JAK2/STAT3 signaling pathway. Our data demonstrated that LPS ICV injection activated hepcidin mRNA expression not only in the brain but also in the peripheral areas of the liver. This finding implies that CNS inflammation also mediates peripheral iron metabolism.

LPS is a bacterial endotoxin and primarily triggers an inflammatory response. Systemic inflammation in an animal model is induced with LPS by IP or intravenous (IV) injection. However, peripheral animal models with LPS treatment are also widely used to induce neuro-inflammation (31). It has been demonstrated that macrophages and glia cells, as well as pro-inflammatory cytokines, were activated and induced by peripheral and CNS administration of LPS (32, 33). Several studies have used LPS by IP or IV injection at various doses ranging from 0.02 to 3 mg/kg body weight, and it was reported that the elevation of hepcidin mRNA expression in the liver was about 2–3-fold, 4 or 6 h after mice were administered with IP injection of LPS (0.5 mg/kg or 1 μg/g body weight) (34, 35). The increase in hepatic hepcidin and IL-6 mRNA at 1.5-fold and 100-fold, respectively, has also been observed in mice 6 h after a single 2 mg/kg administration of LPS IP injection (36). In our present study, a single dose of 5 μg in 2 μL saline of LPS was administered into the cerebral lateral ventricle. Our data have shown that hepcidin mRNA expression in the liver of IL-6+/+ mice increased up to 2.5-fold compared with that in the control mice with PBS injection, and the elevation of IL-6 mRNA in the liver was almost 150-fold after LPS ICV injection. These data implied that LPS had no effect on hepcidin mRNA expression when injected into the brain, and this does not differ from the effect of LPS with IP injection at a similar dose and time-point. However, LPS is a macromolecule that is unable to cross the BBB to regulate hepcidin expression in the peripheral tissues directly (37). We speculated that the inflammatory response induced by LPS injection in the brain generated auto-amplificatory loops after the activation of glia cells in the brain, and the production of IL-6 and other pro-inflammatory cytokines induced by LPS in the brain changed the permeability of BBB, resulting in the leakage of LPS and IL-6 into peripheral circulation. The increased level of IL-6 in circulation also induced the expression of hepatic hepcidin mRNA. However, this hypothesis requires further investigation.

The increased hepcidin synthesis decreased Fpn1 expression in the liver by binding to and inducing the degradation of Fpn1 in the enterocytes of the duodenum, leading to a decrease of serum iron (13). Therefore, serum iron level and transferrin saturation after LPS ICV injection were also investigated. Our data showed that IL-6+/+ mice displayed a significant decrease in serum iron and transferrin saturation 6 h after LPS administration. However, IL-6–/– mice significantly inhibited the decrease of these two parameters. Serum iron and transferrin saturation after LPS ICV injection were slightly lower than those after PBS treatment in IL-6–/– mice, with no significant difference. These results might be due to the partly eliminated hepatic hepcidin expression as shown in Figure 1A. Our findings indicate that IL-6 plays a crucial role in regulating hepcidin expression and other molecules or that inflammatory cytokines are partly involved in hepcidin expression as well.

It is known that LPS induces hepcidin expression through the IL-6/JAK2/STAT3 signaling pathway (25). We demonstrated the role of the IL-6/JAK2/STAT3 signaling pathway and the phosphorylation of STAT3 in the liver. Our results showed a significantly increased expression of p-STAT3 protein in the liver. As mentioned above, LPS triggers various pro-inflammatory cytokines that induce hepcidin expression. However, our present study was performed on IL-6 knockout mice and mainly focused on the IL-6/JAK2/STAT3 signaling pathway induced by LPS after its ICV injection. Other mechanisms need to be explored to fully understand the regulation of hepatic hepcidin expression after LPS ICV injection. To investigate serum iron reduction by hepcidin, the levels of the only known iron exporter Fpn1 and iron storage protein Ft-L were examined. Our data showed that hepatic Fpn1 expression did not change after PBS administration in IL-6–/– mice compared with that in IL-6+/+ mice. This finding was consistent with hepcidin mRNA and p-STAT3 protein expression levels in these two groups. However, our previous study has shown that Fpn1 decreased significantly in IL-6–/– mice compared with IL-6+/+ mice with PBS treatment (28). Based on the different observation in Fpn1 expression, we proposed that the distinct effect of IL-6 on Fpn1 expression in the liver and the brain caused this difference, and the relevant mechanisms are currently unknown.

AG490 is a compound that inhibits tyrosine kinase and the inhibitor of the JAK2/STAT3 signaling pathway. *In vivo* and *in vitro* studies have demonstrated that AG490 could significantly inhibit hepcidin expression in the hepatocyte mice model (38, 39). AG490 exerts an inhibitory effect on hepcidin expression by inhibiting the phosphorylation of STAT3 when exposed to IL-6 (40). Therefore, in our present study, AG490 was selected to inhibit the IL-6/JAK2/STAT3 signaling pathway and to verify the role of this pathway in hepcidin expression. Our previous study has investigated the effect of stattic on hepcidin expression (28), another direct inhibitor of p-STAT3 expression. Both the previous and present studies demonstrate that LPS ICV injection activates the IL-6/JAK2/STAT3 pathway, subsequently inducing hepcidin expression in the brain and the liver.

However, our results should be viewed with certain limitations, specifically the small sample size. We only detected the Fpn1 and Ft-L proteins; other proteins participating in iron metabolism should be included in future larger studies.

In conclusion, our present *in vivo* study showed that LPS ICV injection elevated hepatic hepcidin expression partly through IL-6/JAK2/STAT3 pathway; this research indicated that central inflammation could affect the peripheral iron metabolism through influencing Fpn1 and Ft-L protein. Our study suggested that central inflammation could affect peripheral inflammation and the consequences for iron metabolism. The deeper mechanistic explanations are currently unknown, and further investigations are needed.

## Data Availability Statement

The raw data supporting the conclusions of this article will be made available by the authors, without undue reservation.

## Ethics Statement

The animal study was reviewed and approved by The Ministry of Health of the People's Republic of China and approved by the Animal Ethics Committees of Fudan University, Shanghai, China.

## Author Contributions

MZ and ZQ conceived, organized, supervised the study, drafted, and revised the manuscript. FZ performed the ICV injection, real-time PCR, western blot, serum iron and transferrin saturation assay, and ELISA. PZ analyzed the data. All authors contributed to the article and approved the submitted version.

## Conflict of Interest

The authors declare that the research was conducted in the absence of any commercial or financial relationships that could be construed as a potential conflict of interest.
